# Impact of geriatric consultation teams on clinical outcome in acute hospitals: a systematic review and meta-analysis

**DOI:** 10.1186/1741-7015-11-48

**Published:** 2013-02-22

**Authors:** Mieke Deschodt, Johan Flamaing, Patrick Haentjens, Steven Boonen, Koen Milisen

**Affiliations:** 1 Center for Health Services and Nursing Research, KU Leuven, Kapucijnenvoer 35/4, 3000 Leuven, Belgium; 2 Division of Geriatric Medicine, University Hospitals Leuven, Belgium, Herestraat 49, 3000 Leuven, Belgium; 3 Center for Outcomes Research, Laboratory for Experimental Surgery, Universitair Ziekenhuis Brussel, Vrije Universiteit Brussel, Laarbeeklaan 101, 1090 Brussel, Belgium; 4 Center for Metabolic Bone Diseases, University Hospitals Leuven, Herestraat 49, Leuven, 3000, Belgium

**Keywords:** Acute care, functional status, geriatric consultation, meta-analysis, systematic review

## Abstract

**Background:**

Comprehensive geriatric assessment for older patients admitted to dedicated wards has proven to be beneficial, but the impact of comprehensive geriatric assessment delivered by mobile inpatient geriatric consultation teams remains unclear. This review and meta-analysis aims to determine the impact of inpatient geriatric consultation teams on clinical outcomes of interest in older adults.

**Methods:**

An electronic search of Medline, CINAHL, EMBASE, Web of Science and Invert for English, French and Dutch articles was performed from inception to June 2012. Three independent reviewers selected prospective cohort studies assessing functional status, readmission rate, mortality or length of stay in adults aged 60 years or older. Twelve studies evaluating 4,546 participants in six countries were identified. Methodological quality of the included studies was assessed with the Methodological Index for Non-Randomized Studies.

**Results:**

The individual studies show that an inpatient geriatric consultation team intervention has favorable effects on functional status, readmission and mortality rate. None of the studies found an effect on the length of the hospital stay. The meta-analysis found a beneficial effect of the intervention with regard to mortality rate at 6 months (relative risk 0.66; 95% confidence interval 0.52 to 0.85) and 8 months (relative risk 0.51; confidence interval 0.31 to 0.85) after hospital discharge.

**Conclusions:**

Inpatient geriatric consultation team interventions have a significant impact on mortality rate at 6 and 8 months postdischarge, but have no significant impact on functional status, readmission or length of stay. The reason for the lack of effect on these latter outcomes may be due to insufficient statistical power or the insensitivity of the measuring method for, for example, functional status. The questions of to whom IGCT intervention should be targeted and what can be achieved remain unanswered and require further research.

**Trial registration: **CRD42011001420 (http://www.crd.york.ac.uk/PROSPERO)

## Introduction

The proportion of older persons admitted to the hospital is increasing as a result of the aging of the population. To address the complex needs of frail hospitalized older persons, two types of geriatric assessment models have been introduced, both based on the method of 'comprehensive geriatric assessment' (CGA), defined by Rubenstein *et al*. as 'a multidimensional interdisciplinary diagnostic process focused on determining a frail older person's medical, psychological and functional capability in order to develop a coordinated and integrated plan for treatment and long term follow up' [1,2]. The first type of CGA application is the CGA ward model. Frail older patients are being admitted to a specialized ward where they are under the constant supervision of a specialized multidisciplinary team with geriatric expertise and experience. This model of care is often referred to as the geriatric evaluation and management unit - which includes both acute care as well as inpatient rehabilitation care programs - or the acute care for the elderly unit. It includes the following four components: a specialized environment, patient-centered care, medical review, and interdisciplinary care [3]. A number of reviews and meta-analyses have confirmed the beneficial effects of CGA on wards, and the CGA ward model is accepted as a proven concept [1,4-9]. The clinical value of the second type of CGA, the 'CGA team model', remains unclear. Within this model, frail older patients are hospitalized on a nongeriatric ward - based on the patient's main medical reason for admission - and evaluated by 'a multidisciplinary team which assesses, discusses, and recommends a plan of treatment for frail older inpatients'. This CGA model is typically referred to as the inpatient geriatric consultation team (IGCT), inpatient geriatric consultation service, geriatric assessment team or geriatric liaison team. The concept was initially described and roughly tested by Campion and colleagues in the early eighties [10]. Consultation programs are considered both a component of a chain of geriatric care services and a less expensive substitute for the expensive ward-based model. With acute geriatric units increasingly tested to the limits of their capacity, IGCTs are attractive because they can reach a large number of vulnerable patients and can be implemented in a short period of time [11]. Geriatric consultation services are not only a component of complex geriatric care but also a main organizational principle for organizing hospital care in older patients. Given the shortness of geriatric specialists in Western countries, consultation programs might be a way to bring in geriatrics expertise in an efficient way. More recently, the CGA team model has been implemented in a number of countries (mainly European) as an additional care model to improve quality of care for all geriatric patients hospitalized on nongeriatric wards.

To the best of our knowledge, three reviews have evaluated the effectiveness of IGCTs [1,8,11]. The review by Winograd and Stearns was not done in a systematic way, and included both experimental and nonexperimental studies [11]. Stuck *et al*. performed a meta-analysis of controlled trials reporting on the effects of comprehensive geriatric assessment [8]. No effects were reported on mortality, living location or physical function, but a beneficial effect for IGCT was reported on cognitive function.

In 2011, a Cochrane review [1] reported positive effects for the CGA ward model (for example, increased likelihood of being alive and in their home at up to 12 months after discharge), but no convincing effect of the IGCT model in patients on general, nongeriatric wards was found. However, the meta-analysis did not use the trim-and-fill method to explore the potential impact of publication bias, only randomized controlled trials were included, and potentially important data from nonrandomized controlled studies were not taken into consideration.

In this context, we performed a systematic review and meta-analysis to assess the clinical impact of the CGA team model, based on randomized as well as nonrandomized controlled studies. Our aim was to analyze the effectiveness of an IGCT intervention on functional status, mortality, readmission and length of stay in older patients admitted to the hospital.

## Methods

### Data sources and searches

First, a search for relevant studies was conducted in the databases Medline, CINAHL, EMBASE, Web of Science and Invert, published from inception to June 2012. Keywords for the search were: patient care team (Medical Subject Heading (MeSH)), geriatric care team, geriatric consultation team, consultation team, geriatric evaluation team, geriatric assessment team, geriatric support team, geriatric management team, liaison team, referral and consultation (MeSH), geriatric assessment (MeSH), comprehensive geriatric assessment, CGA, multidisciplinary care, and interdisciplinary care. Subsequently, the reference lists of all relevant studies were searched for additional studies. The review protocol was registered in the International Prospective Register of Systematic Reviews, PROSPERO (CRD42011001420).

### Study selection

Included studies had to be published in English, French or Dutch, and had to meet the following criteria: prospective controlled design (randomized controlled trial or controlled study with parallel controls), a study population of older inpatients (≥60 years and mean age of the study sample of at least 75 years) hospitalized for at least 48 hours on a nongeriatric ward, and measurement of at least one of the following outcomes: functional performance, mortality, readmission, or length of stay. Because an IGCT intervention is a complex intervention [12], it is not always feasible to randomize patients into control and intervention groups for practical reasons (for example, bed capacity per ward) or because of methodological concerns (for example, risk of contamination when control and intervention patients remain in the same ward and are treated by the same staff) [13]. We therefore did not *a priori *exclude nonrandomized studies but explored it as an explanatory variable that may explain discrepancies between individual studies [14].

Reviews were not included, but were hand-searched for relevant references. Grey literature such as conference proceedings, reports and other non peer-reviewed research was not included.

Studies had to clearly describe the method of CGA used. Studies targeting one diagnosis (for example, delirium prevention programs) were excluded from the review. The multidisciplinary team executing the assessment had to consist of at least three different health care disciplines (for example, geriatrician, nurse, social worker, physiotherapist, occupational therapist). After the assessment, the multidisciplinary team had to feed back its recommendations, without being in control of patient management. Studies describing the effect of palliative, psychiatric and psychogeriatric consultation teams, and studies including patients admitted to dedicated geriatric wards were not included. Study selection was done by three independent reviewers (MD, JF and KM). Differences in study selection were discussed until consensus was reached.

### Data extraction and quality assessment

Data of the included studies was extracted by two independent reviewers (MD and PH), based on published information only without contacting researchers to collect additional data. Assessment of the methodological quality of the studies was based on the Methodological Index for Non-Randomized Studies, which consists of 12 criteria [15] (Table 1). Three authors (MD, JF and KM) independently scored all of these criteria on a scale ranging from 0 to 2, depending on whether the criterion was not reported (0), reported but inadequate (1), or reported and adequate (2). We added the criterion 'randomization' as both randomized and nonrandomized controlled studies were included, and scored 0 for nonrandomized studies and 2 for randomized studies. Scoring differences were discussed until consensus was reached. The total quality score ranges from 0 (low quality) to 26 (high quality).

**Table 1:** Methodological quality assessment of the included studies

Study	Clearly stated aim	Inclusion of consecutive patients	Prospective collection of data	Endpoints appropriate to aim of study + ITT	Unbiased assessment of study endpoint(s)	Follow-up period appropriate to aim of study	Loss to follow-up <5%	Prospective calculation of study size	Adequate control group	Con-temporary groups	Baseline equivalence of groups	Adequate statistical analyses	Randomization^a^	Total score
[19]	2	2	2	2	0	2	2	2	2	2	2	2	0	22
[20]	2	2	2	1	2	2	1	0	2	2	1	2	0	19
[21]	2	2	2	1	1	2	0	1	2	2	2	2	2	21
[22]	2	2	2	1	1	2	2	0	2	2	2	2	0	20
[23]	2	2	2	2	1	2	1	2	2	2	2	2	2	23
[25]	2	1	2	1	2	2	2	0	2	2	2	2	2	22
[26]	2	1	2	2	0	2	1	2	0	2	1	2	2	19
[24,27]	2	2	2	1	0	2	1	1	2	2	2	2	2	21
[28,29]	2	2	2	2	2	2	1	2	2	2	2	2	2	25
[30]	2	2	2	1	0	2	1	0	2	2	2	1	2	19
[31]	2	2	2	1	2	2	0	2	2	2	2	2	2	23
[32]	2	2	2	1	2	2	2	1	2	2	2	2	2	24

0 = not reported; 1 = reported but inadequate; 2 = reported and adequate; ^a ^0 for nonrandomized studies and 2 for randomized studies; ITT: intention-to-treat.

### Data synthesis and analysis

The characteristics and effectiveness of the intervention programs are presented in Table 2 and Table 3, respectively. The primary outcome of interest was functional status. Secondary outcomes were all-cause mortality, readmission and length of hospital stay. When data on functional status, all-cause mortality and readmission were available at different time points after discharge, each time point was analyzed separately.

**Table 2:** Setting and study characteristics of the included studies

Study	Study period	Design	Setting	Population	IGCT members	Sample size		Mean age (SD)	
						Intervention	Control	Intervention	Control
[19]	2007 to 2008	CD	Leuven, Belgium	≥65 years with traumatic hip fracture surgery	Geriatrician, geriatric nurse, SW, OT, PT	94	77	80.4 (7.0)	81.1 (7.2)
[20]	1982 to 1984	CD	Montreal, Canada	≥70 years, admitted from ED to medical ward	Geriatrician, geriatric nurse consultant, SW, OT, PT	222	182	78.7 (6.5)	78.3 (6.7)
[21]	1984	RCT	Halifax, Canada	≥75 years, at least one of following criteria: confusion, impaired mobility, falls, urinary incontinence, polypharmacy, living in nursing home, admission within the past 3 months	Geriatrician, nurse, PT	57	56	82.2 (6.2)	83.3 (6.0)
[22]	1985	CD	Halifax, Canada	≥75 years, in category 3 to 5 of Geriatric Status Scale	Specialist in geriatric medicine, nurse coordinator, SW, OT, PT, dietician, pastoral care	66	66	83.0 (5.8)	83.5 (5.9)
[23]	1997 to 2000	Multicenter RCT	Germany	≥65 years with expected LOS of at least 8 days, functionally impaired	Physician, nurse, SW	150	129	79.0 (6.9)	78.4 (6.9)
[25]	1991	RCT	Chicago, USA	≥70 years, admitted from the ED to the medicine service	Geriatrician, SW, medical house staff	51	60	80.1 (6.6)	80.1 (6.7)
[26]	1991 to 1994	RCT	Southern California, USA	≥65 years, at least one of 13 screening criteria (stroke, immobility, impairment in ADL, malnutrition, incontinence, confusion or dementia, prolonged bed rest, falls, depression, social problems, unplanned admission within past 3 months, new fracture, ≥80 years)	Geriatrician, nurse practitioner, SW	1337	1016	77.6 (-)	76.7 (-)
[24,27]	1983 to 1984	RCT	North Carolina, USA	≥75 years admitted to all in-patient units other than intensive care	Attending physician and fellow in geriatric medicine, geriatric CNS, SW	93	92	80.9 (5.8)	82.0 (5.8)
[28,29]	2001 to 2003	RCT	Taiwan	≥60 years with accidental single-side hip fracture	Geriatrician, geriatric nurse, PT, rehabilitation physician	80	82	77.4 (8.2)	78.9 (7.3)
[30]	?	RCT	North Carolina, USA	≥70 years	Physician, CNS, home health nurse, SW, PT, dietician, pharmacist	62	58	77 (5.4)	76 (5.4)
[31]	1997	RCT	Madrid, Spain	≥65 years with acute hip fracture	Geriatrician, SW, rehabilitation specialist	155	164	81.1 (7.8)	82.6 (7.4)
[32]	1985 to 1989	RCT	California, USA	≥65 years, male, functionally impaired and one of the following criteria: confusion, dependence in ADL, polypharmacy, chronic illness, stressed caregiver system	Attending faculty geriatrician, geriatric fellow, CNS, SW, internal medical house officer	99	98	75.7 (9.0)	76.6 (9.7)

ADL: activities of daily living; CD: controlled design; CNS: clinical nurse specialist; ED: emergency department; IGCT: inpatient geriatric consultation team; LOS: length of stay; OT: occupational therapist; PT: physical therapist; RCT: randomized controlled trial; SW: social worker.

**Table 3:** Summary of effectiveness

Study		Follow up					
	Functional status						
	Instrument	Discharge	1 month	3 months	6 months	8 months	12 months
[19]	Six-item Katz ADL Mean (SD) (range 6 to 18)^a^		I: 11.2 (3.4) C: 11.9 (3.1)	I: 10.0 (3.8) C: 10.8 (3.9)			I: 9.8 (3.8) C: 10.0 (3.4)
[20]	Barthel Index Mean (SD) (range 0 to 100)^b^		I: 71.6 (32.6) C: 68.2 (35.8)	I: 80.2 (27.3) C: 75.0 (33.2)	I: 83.1 (26.0) C: 81.7 (28.5)		
[21]	Barthel Index Increase^b^	I: 85% C: 76%					
[22]	Barthel Index Increase			I: 69% C: 52%	I: 77% C: 69%		**I: 75%** **C: 44%**
[23]	Barthel Index Median (IQR) (range 0 to 100)^b^						I: 90 (35) C: 95 (35)
[26]	Basic ADL Mean (95% CI) (range 0 to 100)^b^				I: 80.5 (78.9 to 82.1) C: 80.2 (78.4 to 82.0)		
[24,27]	Seven-item ADL	Better I: 30/88 (34%) C: 23/90 (26%) Same I: 33/88 (38%) C: 35/90 (39%) Worse I: 25/88 (28%) C: 32/90 (36%)					
[28,29]	Chinese Barthel Index Mean (SD) (range 0 to 100)^b^		**I: 81.2 (15.5)** **C: 72.9 (19.8)**	**I: 88.8 (13.4)** **C: 79.9 (20.0)**	**I: 91.8 (11.4)** **C: 84.1 (18.7)**		**I: 90.5 (18.4)** **C: 84.4 (24.0)**
[30]	Functional Assessment Inventory Mean (SD) (range 6 to 30)^a^				I: 14.0 (3.0) C: 14.3 (3.5)		
[31]	Katz ADL recovery (= same or better)	I: 2% C: 0.7%		I: 66/125 (53%) C: 52/125 (43%)			
[32]	Physical Self-Maintenance Score Mean (SD) (range 0 to 6)^a^	I: 3.2 (1.9) C: 3.1 (2.2)		I: 3.4 (2.0) C: 3.6 (2.1)	I: 3.4 (2.0) C: 3.5 (2.2)		I: 3.6 (2.0) C: 4.0 (2.1)

	**Length of stay**		**Mortality**				
			**1 month**	**3/4 months**	**6 months**	**8 months**	**12 months**

[19]	I: mean 11.1 (SD 5.1) C: mean 12.4 (SD 8.5)		I: 4/94 (4.3%) C: 3/77 (3.9%)	I: 15/94 (16.0%) C: 9/77 (11.7%)			I: 20/94 (21.3%) C: 17/77 (22.1%)
[20]	I: mean 20.6 (SD 23.4) C: mean 20.6 (SD 25.3)		I: 20/222 (9.0%) C: 27/182 (14.8%)	I: 45/222 (20.3%) C: 45/182 (24.7%)	I: 56/222 (25.2%) C: 62/182 (34.1%)		
[21]	I: mean 15.8 (SD 12.7) C: mean 14.2 (SD 13.3)			**I: 7/47 (14.9%)** **C: 14/46 (30.4%)**	I: 9/47 (19.1%) C: 14/46 (30.4%)	I: 11/47 (23.4%) C: 18/46 (39.1%)	I: 19/47 (40.4%) C: 20/46 (43.5%)
[22]				**I: 3/56 (5.4%)** **C: 13/56 (23.2%)**	**I: 5/56 (8.9%)** **C: 14/56 (25.0%)**	I: 6/56 (10.7%) C: 16/56 (28.6%)	I: 14/56 (25.0%) C: 20/56 (35.7%)
[23]	I: median 24 (range 18 to 34) C1: median 22 (range 17 to 33)						I: 28/150 (18.7%) C1: 20/129 (15.5%)
[25]	I: mean 5.4 (SD 5.5) C: mean 7.0 (SD 7.0)						
[26]							I: 210/808 (26.0%) C: 155/619 (25.0%)
[24,27]	I: mean 18.3 (SD 16.1) C: mean 16.6 (SD 14.9)				I: 17/86 (19.8%) C: 23/87 (26.4%)		
[28,29]	I: mean 10.1 (SD 3.7) C: mean 9.7 (SD 5.0)		I: 0/80 (0%) C: 0/82 (0%)	I: 3/80 (3.8%) C: 3/82 (3.7%)	I: 6/80 (7.5%) C: 8/82 (9.8%)		I: 13/80 (16.2%) C: 15/82 (18.3%)
[30]	I: mean 9.0 (SD 7.5) C: mean 10.1 (SD 7.6)				**I: 3/62 (4.8%)** **C: 12/58 (20.7%)**		I: 7/68 (10.3%) C: 13/64 (20.3%)
[31]	I: median 16 (IQR 6) C: median18 (IQR 11)						I: 29/154 (18.8%) C: 39/155 (25.2%)
[32]	I: mean 24.8 (SD 22) C: mean 26.7 (SD 33)						I: 41/99 (41.4%) C: 35/98 (35.7%)

			**Readmission**				
			**1 month**	**3 months**	**6 months**	**8 months**	**12 months**

[19]			I: 3/94 (3.2%) C: 5/77 (6.5%)	I: 10/94 (10.6%) C: 11/77 (14.3%)			I: 25/94 (26.6%) C: 22/77 (28.6%)
[22]				I: 10/56 (17.9%) C: 15/56 (26.8%)			I: 23/56 (41.0%) C: 32/56 (56.1%)
[23]							I: 84/150 (56.0%) C: 65/129 (50.4%)
[26]				I: 341/808 (42.2%) C: 278/619 (44.9%)			
[24,27]					I: 29/69 (42.0%) C: 19/64 (29.7%)		
[28,29]			I: 4/80 (5.0%) C: 5/82 (6.1%)	I: 8/80 (10.0%) C: 10/82 (12.2%)	I: 16/80 (20.0%) C: 14/82 (17.1%)		I: 29/80 (36.3%) C: 25/82 (30.5%)
[30]					**I: mean 0.3 (SD 0.6)** **C: mean 0.6 (SD 1.0)**		
[32]							I: mean 1.0 (SD 1.3) C: mean1.2 (SD 1.7)

Figures in bold show a statistically significant difference (*P *≤0.05). ADL: activities of daily living; C: control group; I: intervention group; IQR: interquartile range; NA: not applicable; SD: standard deviation. ^a^The higher the score, the more dependent. ^b^The lower the score, the more dependent.

For each individual study and for each outcome of interest at each time point after discharge, we computed an effect size and its 95% confidence interval (CI). The effect size was the Hedges' g for functional status (continuous variable documented by different scales using different units in the individual studies), the relative risk for all-cause mortality and readmission (binary variables), and the mean difference for length of stay (continuous variable expressed in the same unit (days) in each individual study). Studies reporting an effect of the intervention on mortality on 3 or 4 months follow-up were pooled in one meta-analysis.

For each outcome of interest and at each time point after discharge, the effect sizes of the individual studies were pooled using DerSimonian and Laird random-effects models [16]. Random-effect models assume that the observed variability between the studies and their studied population reflects sampling variability and heterogeneity of the study populations. The results were examined for heterogeneity by visually examining forest plots, by using the Cochran Q test, and by computing the I-squared statistic, with values less than 25% indicating low, 25% to 50% indicating moderate, and values exceeding 50% indicating high heterogeneity. We decided, *a priori*, to explore study design (randomized versus nonrandomized) as a potential source of heterogeneity if the I-squared statistic indicated high heterogeneity (I² greater than 50%). Publication bias was explored visually by the funnel plot method, and assessed by Duval and Tweedie's trim-and-fill method [17]. This method looks for missing studies on the funnel plot and recalculates an adjusted pooled effect size by also including the potentially missed (trimmed) studies. There must be a least three studies published to run this publication bias procedure.

All statistical processes for combining data from multiple studies were done in CMA, version 2 (Comprehensive Meta-Analysis, Biostat TM, Englewood, NJ, USA).

## Results

### Selected studies and methodological quality

**Figure 1:**
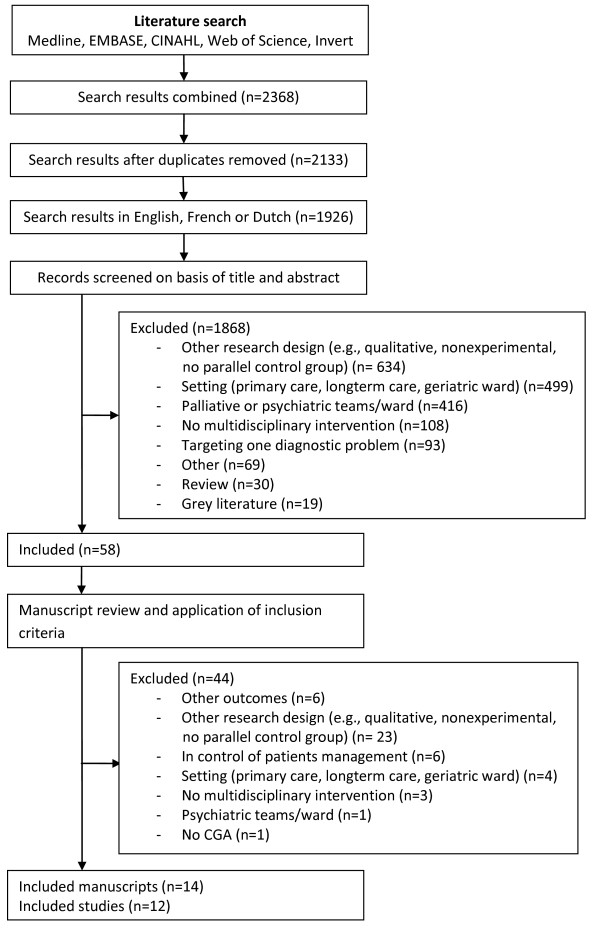
**Flow diagram of the search and selection method **[18].

Figure 1[18] shows a flow diagram of our search and selection process. Fourteen articles were selected based on 12 individual studies [19-32]. The results of the study by Shyu *et al*. were reported in two papers [28,29]. The articles from Saltz *et al*. and McVey *et al*. reported data from the same randomized controlled trial [24,27].

Results of the evaluation of the methodological quality of the studies are shown in Table 1. The total quality scores of the included studies ranged from 19 'moderate' to 25 'excellent' [19-32]. In the majority of the studies, a randomized controlled design was used [21,23-32]. A prospective calculation of the study size was done in only five studies [19,23,26,28,29,31]. The majority of the studies did not perform [21-23] or report [19,24,26,27,30] an unbiased assessment of the study outcomes. In two studies, intervention and control groups were not equivalent at baseline [20,26].

### Characteristics of studies and patients

The selected trials included a total of 4,546 individuals aged 60 years and older (Table 2). The mean age of intervention and control patients at baseline ranged from 75.7 years to 83.5 years. All studies conducted before 1995 were done in the United States [20-22,24-27,30,32] or Canada [20-22], whereas more recent studies were done in Europe [19,23,31] or Taiwan [28,29]. Five studies were done in patients who were frail or with specific geriatric problems [21-23,26,32], whereas three studies were conducted in patients with a recent hip fracture [19,28,29,31]. Four studies had no other inclusion criteria except a minimum age for inclusion [20,24,25,27,30].

### Effects on functional status

The analysis on the effects on functional status was based on 11 trials or 4,435 participants [19-24,26-32] (Table 3). A significant benefit in functional status for intervention patients was found in only two trials. Shyu *et al*. reported better mean scores in the Chinese Barthel Index (range 0 to 100 points) up to one year of follow-up (intervention group: 90.5, SD 18.4 versus control group: 84.4 points, SD 24.0; *P *= 0.002) [28,29]. Hogan and Fox found that 75% of intervention patients compared with 44% of control patients had an increased Barthel Index at one year of follow-up [22].

**Figure 2:**
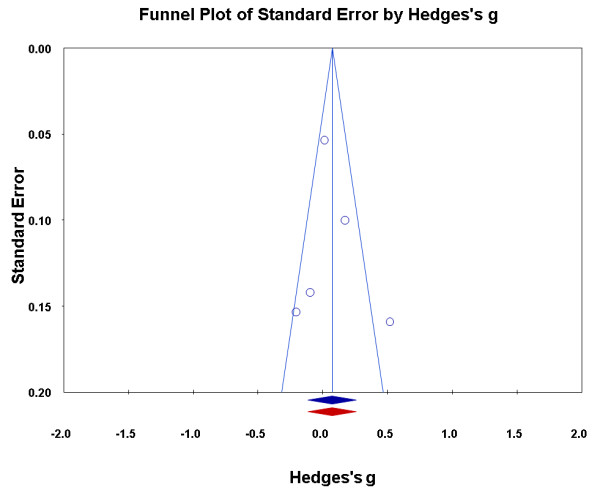
**Publication bias and its potential impact (functional status at 3 months)**. The blue circles represent individual studies, the blue lines are the funnel plot, and the blue diamond is the effect size (Hedges's g) and 95% confidence interval for the current meta-analysis. The red diamond is the effect size (Hedges's g) and 95% confidence interval for the meta-analysis, after adjusting for publication bias.

The random-effects pooled estimates for functional status were very consistent over time, showing no significant effect of the intervention (Table 4). Heterogeneity was statistically significant in all but one time point, but this high heterogeneity could not be explained by study design. The funnel plots were asymmetric, suggesting the absence of or inability to find small studies. Figure 2 shows the funnel plot for functional status at 3 months follow-up. The funnel plots for the other time points were similar (figures not shown). However, all 95% CIs of the adjusted pooled estimates imputed according to the trim-and-fill method were almost identical to those calculated previously, and all included the value of no effect, providing evidence that publication bias is unlikely to have affected our findings, and suggesting that, overall, treatment had no impact on functional status after discharge.

**Table 4:** Meta-analysis

Outcome	Follow-up (months)	Treatment effect			Heterogeneity		Publication bias		Number of studies
			
		Effect measure	Pooled estimate (95% CI)	*P *value for treatment effect	*P *value for Cochran's Q test	I squared statistic^a^	Number of studies trimmed^b^	Adjusted pooled estimate^c^	
Functional status	1	Hedges' g	0.11 (-0.22 to 0.45)	0.50	0.009	79%	0	0.11 (-0.22 to 0.45)	3 [19,20,28,29]
	3		0.07 (-0.11 to 0.26)	0.44	0.006	73%	0	0.07 (-0.11 to 0.26)	5 [19,20,28,29,31,32]
	6		0.09 (-0.14 to 0.33)	0.41	0.034	63%	1	0.15 (-0.07 to 0.37)	5 [20,26,28-30,32]
	12		0.01 (-0.15 to 0.16)	0.94	0.15	44%	0	0.01 (-0.15 to 0.16)	4 [19,23,28,29,32]
Length of stay		Weighted mean difference	-0.35 (-1.24 to 0.55)	0.45	0.75	0%	1	-0.34 (-1.24 to 0.56)	9 [19-21,24,25,27-32]
Mortality	1	Relative risk	0.66 (0.40 to 1.09)	0.10	0.46	0%	NA	NA	2 [19,20]
	3/4		0.72 (0.44 to 1.17)	0.19	0.12	47%	0	0.72 (0.44 to 1.17)	5[19-22,28,29]
	6		**0.66 (0.52 to 0.85)**	**0.001**	0.37	0%	2	0.73 (0.52 to 1.02)	6 [20-22,24,27-30]
	8		**0.51 (0.31 to 0.85)**	**0.009**	0.39	0%	NA	NA	2 [21,22]
	12		0.98 (0.86 to 1.11)	0.75	0.52	0%	2	1.01 (0.87 to 1.16)	9 [19,21-23,26,28-32]
Readmission	1	Relative risk	0.65 (0.25 to 1.67)	0.37	0.59	0%	NA	NA	2 [19,28,29]
	3		0.93 (0.82 to 1.04)	0.18	0.74	0%	2	0.94 (0.84 to 1.05)	4 [19,22,26,28,29]
	6		0.88 (0.40 to 1.96)	0.76	0.001	86%	0	0.88 (0.40 to 1.96)	3 [24,27-30]
	12		0.99 (0.83 to 1.19)	0.94	0.34	12%	2	1.09 (0.87 to 1.36)	5 [19,22,23,28,32]

CI: confidence interval; NA: not applicable. ^a^I squared statistic: values less than 25% indicate low, 25% to 50% indicate moderate, and greater than 50% indicate high heterogeneity. ^b^There must be a least three studies published to run publication bias procedure. ^c^Adjusted pooled estimate according to Duval and Tweedie's trim-and-fill analysis.

### Effects on length of stay

The analysis on the effects on length of stay was based on 10 trials or 2,061 participants [19-21,23-25,27-32] (Table 3). None of the trials reported statistically significant differences between the length of stay of the intervention and control groups. Large variation was found in the different studies with regard to the mean length of stay for intervention patients: Naughton *et al*. reported a mean length of stay of 5.4 days (SD 5.5 days), whereas Winograd *et al*. found a mean length of stay of 24.8 days (SD 22 days) [25,32].

The results of the meta-analysis also showed that IGCT intervention was not associated with a difference in length of stay (Table 4). The funnel plots (figures not shown) were asymmetric, but the imputed (adjusted) overall pooled estimate and its 95% CI were almost identical.

### Effects on mortality

Data to study the effect on mortality were available for 11 trials or 4,435 participants (Table 3) [19-24,26-32]. In most studies, an IGCT intervention did not have a significant effect on mortality at any specific time point [19,20,23,24,26,29,31,32]. However, Hogan and Fox reported significantly lower death rates in favor of the intervention group at four months after hospital discharge [22]. The studies of Hogan and Fox and Thomas *et al*. respectively showed that an IGCT intervention resulted in a significant decrease of mortality of 16% and 15% at 6 months after discharge [21,30].

The random-effects pooled estimates for all-cause mortality yielded discordant results depending on time after discharge (Table 4). More specifically, pooled risk estimates showed a beneficial effect of the intervention at 6 months and 8 months, while a noneffect was found at 1 month, 3 months and 1 year after discharge. The high heterogeneity at 3 months (I² = 47%) was not related to study design.

### Effects on readmission

To study the effect on readmission, data were available for eight trials or 3,599 participants (Table 3) [19,22-24,26-30,32]. Only two trials reported statistically significant differences for effects on readmission in favor of the intervention group. Thomas *et al*. found fewer readmissions per patient at 6 months follow-up (intervention group: mean 0.3, SD 0.6 versus control group: mean 0.6, SD 1.0), while Kircher *et al*. found fewer readmitted intervention patients at 12 months follow-up compared with the comparison group (intervention group: 84 out of 150 (56%) versus control group: 30 out of 81 (37.0%)) [23,30].

The random-effects pooled estimates for readmission were very consistent over time showing a noneffect of the IGCT intervention (Table 4). The high heterogeneity at 6 months after discharge (I² = 86%) was not related to study design. The funnel plots were asymmetric (figures not shown), but publication bias was unlikely to change our findings that the IGCT intervention has no impact on readmission.

## Discussion

CGA conducted on dedicated geriatric wards has proven to be beneficial for frail older patients [1,4,6-9], but the effectiveness of IGCTs remains unclear. In the current systematic review and meta-analysis, we were unable to document any favorable effect of IGCT interventions on functional status in older hospitalized patients, our primary outcome. Although most studies used the Barthel Index to assess basic activities for daily living, there was a high percentage of heterogeneity at all time points. Furthermore, there was a strong ceiling effect of most of the functional measures used, which is unfortunately a frequent limitation of these types of studies and may have limited the ability to detect improvements over time. Although a significant reduction in mortality rate was found at 6 and 8 months follow-up in intervention patients, this effect was not confirmed at any of the other follow-up points, including the 1-year follow-up, which combined the results of nine individual studies.

No effect of the intervention was found on the readmission rate at 1, 3, 6 and 12 months of follow-up, although it should be mentioned that only one study clearly reported unplanned readmission rate [19]. Because planned readmissions cannot, and probably should not, be prevented, 'avoidable readmission rate' would have been a better outcome measure. However, preventability of readmissions remains an understudied topic [33]. A recent systematic review of 34 studies found wide variation (ranging from 5% to 79%) in the percentage of readmissions considered preventable [34]. There was, however, only one validated prediction model that explicitly examined potentially preventable readmissions as an outcome [35].

IGCT intervention is sometimes perceived by healthcare workers as a strategy that may prolong the patient's length of stay. However, we could not find any effect of IGCT on the length of the hospital stay.

Our analyses come with methodological limitations, the main being the heterogeneous way in which IGCTs are organized and put into practice. Because of the complexity of the interventions, explicit instructions are required to improve the consistency and reproducibility of the intervention. Although all studies reported the use of a formal multidisciplinary CGA in the intervention, the exact composition of the team, the frequency of interdisciplinary meetings, and the frequency of patient visits varied greatly or was not described in sufficient detail in the individual studies. Characteristics of the country (for example, healthcare and insurance system) and the hospital (for example, admission and discharge policy), rarely discussed in the studies, may also have affected the impact of the intervention, especially on outcomes such as length of stay and readmission rate. Additionally, in the study by Shyu *et al*., the geriatric consultation was accompanied by a rehabilitation program that was partly delivered by a geriatric nurse [28,29]. This more elaborate intervention may explain the positive effect on functional status in this particular study, but there remains a lack of effect of the intervention on functional status after meta-analysis.

Because IGCTs constitute an advisory model, another important factor that could explain the limited impact of the intervention may be the lack of adherence to the recommendations made by the IGCT. The overall adherence rate to the IGCT recommendations was only reported in three of the included studies [19,32,36]. The nonadherence rate ranged from 23% to 33%, providing strong evidence that the intervention does not meet its full potential. Having control over the care process is one of the key differences between the two CGA models and could be one of the main reasons why the CGA ward type is effective and the team type (IGCT) is not.

Improved targeting to patients who will benefit most from an IGCT intervention has also been suggested to make this type of intervention more effective [37]. Unfortunately, the extent to which patient characteristics contribute to the outcome of IGCT intervention could not be assessed because of the small number of studies included, precluding subgroup meta-analyses. Commonly used screening tools, like the Triage Risk Screening Tool [38] or the Identification for Seniors at Risk [39], might be helpful in identifying patients at the greatest risk for functional decline, but these instruments are limited by low specificity and low positive predictive value [40,41]. This results in a high number of false-positives and the investment of a significant amount of time and manpower in older persons unlikely to benefit from IGCT in-depth comprehensive geriatric assessment or interventions.

Despite the methodological quality of the included studies ranging from moderate to good, they all performed poorly with regard to blinding of patients and/or assessment team. It is difficult to meet all criteria with high methodological quality in this research area - that is, complex multidisciplinary interventions with face-to-face contact. This is a further limitation that hampers progress [13].

Finally, we acknowledge that only a limited number of major outcomes was studied in our analysis; other outcomes must also be considered in the discussion of an IGCT. For example, in four studies, an IGCT intervention had a significant impact on cognitive outcome, such as incidence of delirium or improvements in the Mini-Mental State Exam or the Geriatric Depression Scale [21,28,32,42]. Among other outcomes, cognitive status is a clinically important indicator that should be considered in further studies.

## Conclusions

A systematic review and meta-analyses of randomized and nonrandomized studies in older people hospitalized on nongeriatric wards could not show a significant impact of an IGCT intervention on functional status, readmission or length of stay. At 6 and 8 months follow-up, significantly fewer intervention patients had died, but the effect on mortality at the other time points was not significant. The lack of control over the implementation of proposed interventions is likely to be one of the main limitations of this type of advisory care. The questions of to whom IGCT intervention should be targeted and what can be achieved remain unanswered and require further research.

## Abbreviations

CGA: comprehensive geriatric assessment; CI: confidence interval; IGCT: inpatient geriatric consultation team; MeSH: Medical Subject Headings; SD: standard deviation.

## Competing interests

The authors declare that they have no competing interests.

## Authors' contributions

MD was responsible for the study concept and design, the selection of the studies and a critical appraisal of included studies, the data extraction, interpretation of data, and drafting the manuscript. JF participated in the study concept and design, the selection of studies and the critical appraisal of included studies, and the interpretation of data. PH participated in the data extraction, was responsible for statistical analysis and participated in the interpretation of data. SB participated in the study concept and design, and the interpretation of data. KM was responsible for the study concept and design, the selection of studies and the critical appraisal of included studies, interpretation of data, and drafting the manuscript. All authors read, revised and approved the final manuscript. Supervision was done by KM and JF.

## Pre-publication history

The pre-publication history for this paper can be accessed here:

http://www.biomedcentral.com/1741-7015/11/48/prepub
